# Hydrothermal Conversion of Spent Sugar Beets into High-Value Platform Molecules

**DOI:** 10.3390/molecules25173914

**Published:** 2020-08-27

**Authors:** Jens Pfersich, Pablo J. Arauzo, Michela Lucian, Pierpaolo Modugno, Maria-Magdalena Titirici, Luca Fiori, Andrea Kruse

**Affiliations:** 1 Conversion Technologies of Biobased Resources, University of Hohenheim, Garbenstrasse 9, 70599 Stuttgart, Germany; Pabloj.Arauzo@uni-hohenheim.de (P.J.A.); Andrea_Kruse@uni-hohenheim.de (A.K.); 2 Department of Civil, Environmental and Mechanical Engineering, University of Trento, Via Mesiano 77, 38123 Trento, Italy; Michela.Lucian@unitn.it (M.L.); Luca.Fiori@unitn.it (L.F.); 3 School of Engineering and Materials Science, Queen Mary University of London, Mile End Road, London E1 4NS, UK; P.Modugno@qmul.ac.uk; 4 Department of Chemical Engineering, Imperial College London, South Kensington, London SW7 2AZ, UK; M.Titirici@imperial.ac.uk

**Keywords:** agro-residues, sugar beets, biomass, hydrothermal carbonization, hydrolysis, sugars, HMF, hydrochar, biorefinery

## Abstract

The growing importance of bio-based products, combined with the desire to decrease the production of wastes, boosts the necessity to use wastes as raw materials for bio-based products. A waste material with a large potential is spent sugar beets, which are mainly used as animal feeds or fertilizers. After hydrothermal treatment, the produced chars exhibited an H/C ratio of 1.2 and a higher heating value of 22.7 MJ/kg, which were similar to that of subbituminous coal and higher than that of lignite. Moreover, the treatment of 25 g/L of glucose and 22 g/L of fructose by heating up to 160 °C led to a possible application of spent sugar beets for the production of 5-hydroxymethylfurfural. In the present study, the maximum concentration of 5-hydroxymethylfurfural was 3.4 g/L after heating up to 200 °C.

## 1. Introduction

The depletion of current petrochemical reserves to produce energy and fine chemicals, as well as the growth of CO_2_ emissions, which is one of the factors of climate change [1], motivate to find sources of renewable energies. Biomasses like wood, forestry residues, agricultural wastes and urban wastes are the sole known renewable carbon sources with sufficient carbon content [2] and an environment-friendly neutral CO_2_ balance.

Thermochemical conversion of biomass is considered as one of the pathways for the production of renewable energy and valuable chemicals or materials. The study of different thermochemical processes such as pyrolysis, gasification, combustion and hydrothermal carbonization (HTC) are well-illustrated in previous studies with model compounds (i.e., microcrystalline cellulose and glucose) for the production of different products [3,4,5]. On the other hand, the wish to use biomass in the frame of biorefinery still necessitates research to improve the processes and implement different kinds of biomasses. Biorefinery concepts include using complex biomass as feedstock, which contains different amounts and compositions of biopolymers such as hemicellulose, cellulose and lignin. Thus, a comparison of biomasses with model compounds is necessary.

**Figure 1:**
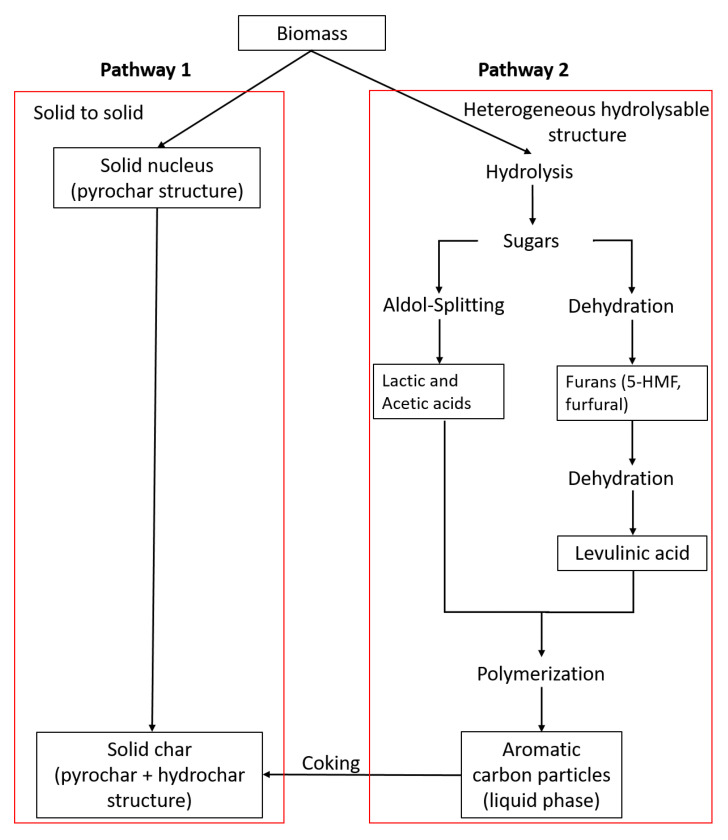
Possible char formation pathways of biomasses (e.g., spent sugar beets (SPB)) during hydrothermal treatment (HT); modified from Kruse et al. and Karaldiyrim [19,20].

One of the thermochemical processes which rose notably during the last years due to its simple integration into biorefinery concepts is the process of hydrothermal conversion. This conversion process is considered to be the most suitable for the conversion of wet biomass to gain valuable solid and liquid products which are rich in valuable compounds like furans, organic acids (acetic and levulinic acids) and sugars (glucose and fructose). The selectivity during the conversion of the initial biomass to obtain these compounds in the liquid phase is mainly affected by the process pH, temperature and reaction time [6,7,8,9]. Moreover, in the present study, the focus was hydrothermal treatment (HT) of the biomass to obtain a solid product as well as intermediates of the hydrolysis step. Thus, it is necessary to understand how to increase the selectivity of the conversion of intermediate products, which results in high-value chemical compounds, by comprehending the reaction routes originating from the biomass via HT (Figure 1). One of the high-value molecules, which can be obtained during hydrothermal treatment, is 5-hydroxymethylfurfural (HMF). HMF consists of a heterocyclic furan ring with a hydroxyl group as well as an aldehyde group [10,11]. This structure is crucial to mainly undergo reduction (e.g., 2,5-*bis*(hydroxymethyl)furan), oxidation (e.g., 2,5-furandicarboxylic acid) and esterification reactions [12,13,14].

Several types of feedstock can be used as raw materials for HMF production: monosaccharides (fructose or glucose), disaccharides (maltose, sucrose or cellobiose) and polysaccharides (starch, inulin or cellulose) [15]. Although fructose is the preferred starting material to obtain optimal HMF yield, it should be clear that biomasses rich in cellulose, the major component in plant biomass, are more suitable for the large-scale production of HMF [16,17,18].

The integration of HMF production in biorefinery concepts requires a suitable starting biomass, which initially contains a high amount of glucose to be converted by hydrothermal treatment. One of the biomasses, which fits this purpose, is sugar beets originally containing 15–20 wt.% sugar [21]. The sugar is extracted during industrial processes, while the rest of the plant serves as a mediocre fertilizer or animal feed [21]. In 2018/2019, Südzucker, a leading sugar production company of Germany, produced 4.6 million tons of sugar from 29.4 million tons of sugar beets [22]. Totally, 25% of the sugar produced worldwide originates from sugar beets [21]. Thus, a high amount of potential feedstock for biorefineries is available, which would be another advantage adding to the short ways between farms and sugar production facilities [23]. After sugar extraction, exhausted sugar beets are called spent sugar beets (SPB).

Therefore, the aim of this study was to evaluate the selectivity and conversion of SPB as an initial feedstock to sugars as a product of HT. Furthermore, intermediate products of the hydrothermal conversion like HMF are discussed. These results are compared with previous studies, which used model compounds to predict the formation of these intermediates.

## 2. Results and Discussion

### 2.1. Feedstock Characterization

Table 1 shows the fiber composition of SPB used for HT tests in this study. It was found that the SPB consists of less cellulose and hemicellulose than raw sugar beets [24]. This difference in composition may be due to the breaking of the original fibers of SPB after sugar production because of thermal treatments (e.g., slicing, cooking and mashing) [25]. In the case of polysaccharides (cellulose and hemicellulose), a higher reduction of the hemicellulose content is obtained compared to cellulose. This is due to the branched chains of hemicellulose and the different monomers and saccharides, which are the building blocks of hemicellulose. While the chains of cellulose can arrange themselves in secondary structures like lamellae and fibers consisting of crystalline areas, thus containing a higher stability against reagent penetration due to its regular structure, hemicellulose is not able to form highly arranged structures [26,27,28]. Side chains and different monomers cause different distances between the hemicellulose chains, which is the reason for weaker and fewer interactions between the chains [26,27,28]. Therefore, the chains of hemicellulose appear as easier targets for water, thus being hydrolyzed faster [26,28]. Nonetheless, this amorphous structure enables hemicellulose chains to form many interactions with cellulose and lignin, leading to a high stability of the plant material. Furthermore, cellulose also shows a different hydrolysis behavior, which is a result of the amorphous and crystalline structures of cellulose [29]. Differences between the raw sugar beets and the spent sugar beets may be caused by a partial hydrolysis of the amorphous part of cellulose during sugar extraction, while the crystalline structure remains mostly unaffected in the SPB. This agrees with previous studies, which showed that the amorphous structure of the cellulose tends to be hydrolyzed faster than the crystalline structure [5,30,31].

### 2.2. Solid Characterization

Table 2 shows the operating conditions of the HT and the physicochemical properties of the obtained solid. The selection of reaction temperatures of 160 °C and 200 °C was based on the research of Titirici et al. [32] and Kruse et al. [33]. Cellulose is completely composed of D-glucose units, while hemicellulose also comprises other sugars. At 160 °C, glucose starts to get converted to hydrochar, which is in agreement with other studies that mention that the conversion of hemicellulose during hydrothermal carbonization starts between 160 °C and 180 °C [32,34,35]. In addition, several studies [32,36,37] showed that, at 200 °C, the cellulose structure starts to get degraded and consequently converted into hydrochar. Moreover, the conversion of biomass into char by hydrothermal treatment can proceed via two different pathways. First, direct solid-to-solid conversion, usually associated with lignin, which is referred to as pyrochar. Second, the degradation of hydrolyzed molecules, derived from the volatile matter (VM), to intermediates like furans with a subsequent polymerization to produce coke particles (often referred to as secondary char) [38,39,40]. Due to the considerably low lignin content in SPB (shown in Table 1), the impact of this first pathway was supposed to be not significant in this study. Hence, the second pathway was considered as the preferred pathway to convert the SBP to hydrochar. As the main field of interest in this study was the conversion of the initial biomass to intermediates, the reaction time started when the reactor reached the reaction temperature. Increasing the operating temperature from 160 °C to 200 °C led to an almost time-independent reduction of VM (Table 2).

Table 2 shows that increasing the reaction time from 0 h to 1 h only led to a slight decrease of the VM and increase of fixed carbon (FC). While the decrease in the VM and the increase in FC at 160 °C were around 4 wt.% by increasing the reaction time to 1 h, the decrement at 200 °C was around 2 wt.% for the same increase in reaction time. Moreover, at 160 °C, the yield of hydrochar decreased with the reaction time, which agreed with previous studies [38,39]. However, at 200 °C, the increasing reaction time leads to a contrary effect as previously observed. Thus, it can be assumed that some of the intermediates, which remained in the process water in the experiments for 0 h and 0.5 h, reacted via a polycondensation mechanism and precipitated to increase the yield of hydrochar.

To confirm this, dissolved organic carbon (DOC) of the liquid samples (Supplementary Information, Table S1) was determined. It was observed that an increase of reaction time led to a reduction of DOC for both temperatures. At 200 °C, this decrease of the intermediates and increase of the yield of hydrochar implied that the conversion of initial VM to intermediates was completed (Table 2). Additionally, at 160 °C, this reduction also implied the formation of coke, often called secondary char. However, the kinetic rate of degradation of the VM was higher than the kinetic rate of formation of coke from intermediates (5-hydroxymethylfurfural and furfural) [39,40,41,42].

**Figure 2:**
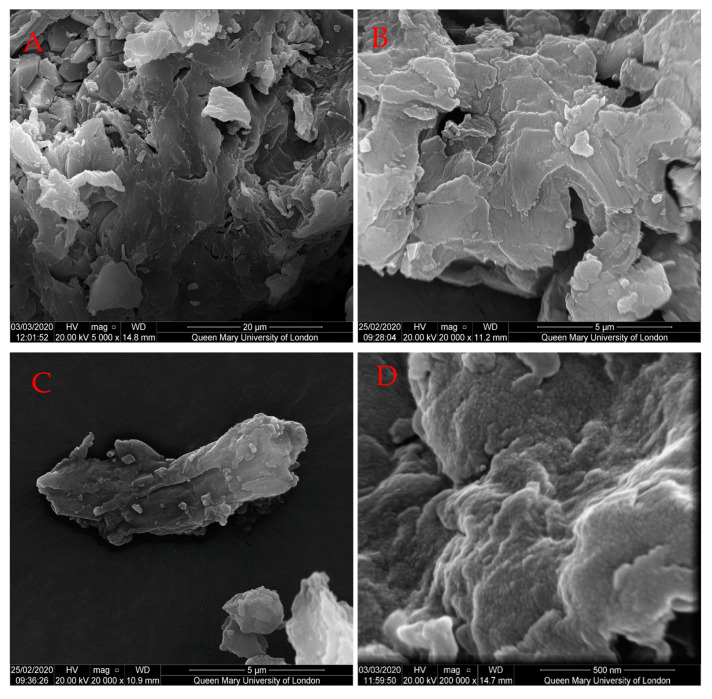

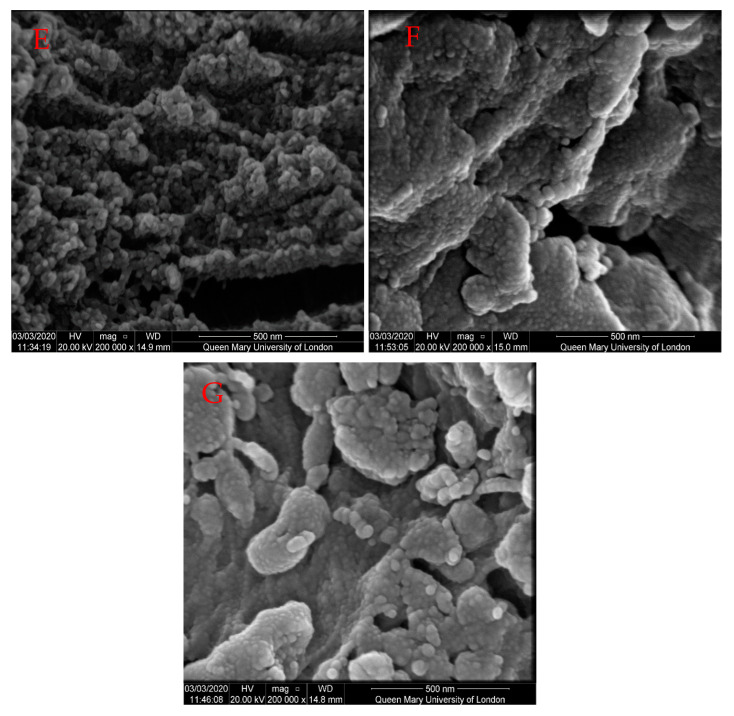
SEM images of raw SPB and chars after different reaction times at different temperatures. (**A**) Raw, (**B**) S_160_0, (**C**) S_160_0.5, (**D**) S_160_1, (**E**) S_200_0, (**F**) S_200_0.5, (**G**) S_200_1.

In this study, a scanning electron microscopy (SEM) analysis was performed to confirm the hypothesis that, mostly the second pathway (formation of hydrochars) takes place [43]. While the raw SPB exhibited a smooth surface and mainly consisted of large pieces, temperature and reaction time showed their impact on the surface of the chars (Figure 2). This uniform surface was changed in two different ways, which included cracking of the huge pieces due to thermal stress and reactions as well as becoming rough because of charring. While cracking of the fibers of the original biomass led to new reaction sites, the charring occurred all over the surface by forming spherical structures. Those spheres were usually considered to be secondary char, the reaction products of polycondensation of molecules derived from hydrolysis, mainly via HMF. At 160 °C, the formation of hydrochar occurred only to a limited degree during the regarded reaction time. Especially for longer reaction times, spherical structures appeared on the surface of the SPB. Contrary to this, at 200 °C, the hydrochar was already formed as the reaction temperature was reached, which was represented by the sphere covered surface at 0 h (Figure 2E) and was completely covered by microspheres. With the passage of time, the number of microspheres increased, eventually forming macrospheres via coagulation (Figure 2G) [44]. An immediate formation of char during the heating phase of the reactor was negligible for 160 °C with respect to the aforementioned production of char to a limited degree. Char formation started between 160 °C and 200 °C due to the degradation of the biopolymers and solid-to-solid conversion. An increase in reaction time also increased the density of the spheres across the surface, finally leading to a thicker layer of secondary char.

What was not visible, even with the SEM, was the inner structure of the char particles. It can be assumed that the forming hydrochar layer covered unreacted biomass, as well as reactions that occurred inside. However, considering the SEM images, the formation of pyrochar was not clearly distinguishable from hydrochar formation due to the high amount of hydrochar covering. It was assumed that the “seeds” were formed by the pyrolysis pathway (Figure 1) around which the hydrothermal carbonization chars were grown [38]. At 200 °C, the temperature was high enough to start pyrochar formation to a comparatively high degree by converting more of the original biomass (decarboxylation and decarbonylation of undissolved biomass), thus leading to the formation of agglomerates and subsequently their precipitation [39]. This was one possible reason for the increased amount of chars in the SEM, especially considering the formation of hydrochar around pyrochar [38]. 

**Figure 3:**
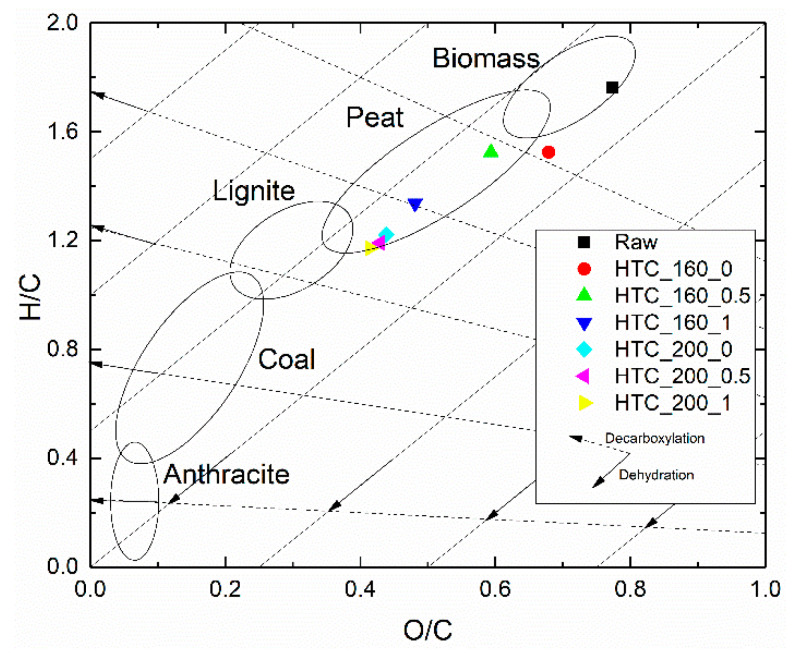
van Krevelen diagram of raw SPB and produced chars.

Figure 3 and Table S3 show the O/C and H/C atomic ratios of the produced hydrochars. As expected, the highest O/C and H/C atomic ratios were obtained for raw SPB (upper right corner of Figure 3). Compared with original SPB, the O/C and H/C atomic ratios of the hydrochars decreased. This decrease in O/C and H/C ratios was mainly due to dehydration and decarboxylation reactions that occurred during the HT process. Higher reaction temperatures and longer reaction times lead to hydrochars with properties similar to sub-bituminous coal, which can be used as a co-fuel [9,45]. By considering the results in literature [46,47,48], the obtained H/C and O/C ratios in Figure 3 were close to that of other solid fuels like coal, lignite and peat, thus showing their possibility to be used as co-fuels.

The higher heating value (HHV) of the hydrochars, produced at high temperatures, was around 22 MJ/kg (Table 3), which was similar to the HHV of HMF (22.06 MJ/kg) [49]. This was in good agreement with the hypothesis that intermediates (e.g., HMF) condensate to form hydrochars [50].

### 2.3. Liquid Characterization

Results of the HPLC analysis are given in Supplementary Information (Tables S1 and S2). The initial pH of SPB was 5.98; however, the pH after hydrolysis of SPB at 160 °C decreased with longer reaction times. This was proof that the hydrolysis pathways were proceeding as explained due to the starting degradation of hemicellulose and consequently beginning the formation of acids (acetic, lactic, levulinic, formic and propionic acids) [52]. At 200 °C, the pH increased with the reaction time, implying the end of the conversion process and probable adsorbtion of formed acids on the chars. The lower concentration of dissolved organic carbon (DOC) at 200 °C confirmed the polymerization of intermediates to secondary char.

Cellulose is a linear polymer with high molecular weight and is composed of D-glucose units linked by β-(1-4) glycosidic bonds [53,54]. In contrast, hemicellulose consists of different sugar monomers and exhibits branched chains. Both biopolymers undergo hydrolysis, but hemicellulose is more reactive because of its amorphous character. Hydrolysis reactions lead to the cleavage of ether bonds of cellulose by the attack of water. For each water molecule consumed, one molecule of glucose is produced. Water, which is an example of a polar protic solvent, also catalyzes the hydrolysis step by offering protons and hydroxyl ions [55]. The catalytic effect of water is more pronounced at higher temperatures and pressure due to its higher ionic product compared with ambient conditions [7,56]. This fact can be emphasized by the studies, which used Brønsted acids (HCl, HBr and H_2_SO_4_) as catalysts [7]. As Brønsted acids offer protons that facilitate the breakdown of C-O-C glycosidic bonds between glucose units, they lead to a faster depolymerization of cellulose [57].

**Figure 4:**
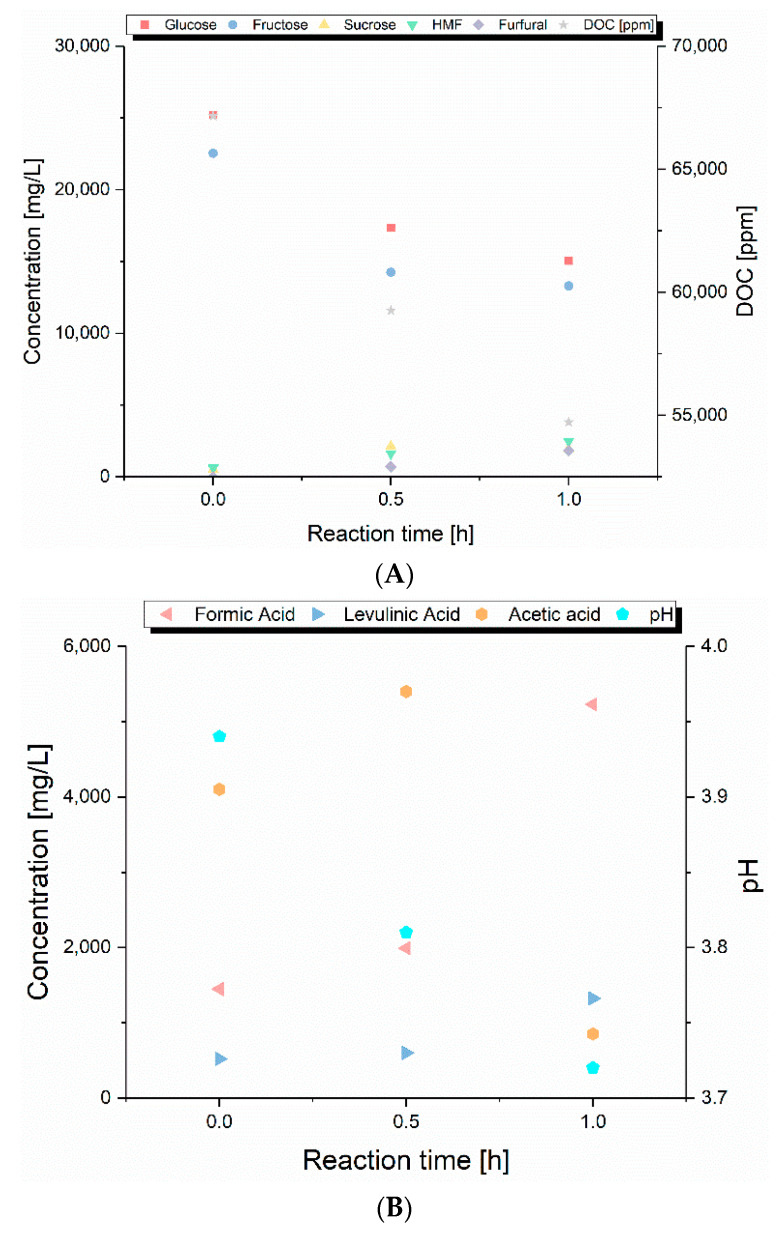
(**A**) Concentration of the sugars, 5-hydroxymethylfurfural (HMF) and furfural as well as dissolved organic carbon (DOC); (**B**) concentration of the acids in the process liquid and their pH after hydrothermal treatment (HT) at 160 °C for different reaction times.

**Figure 5:**
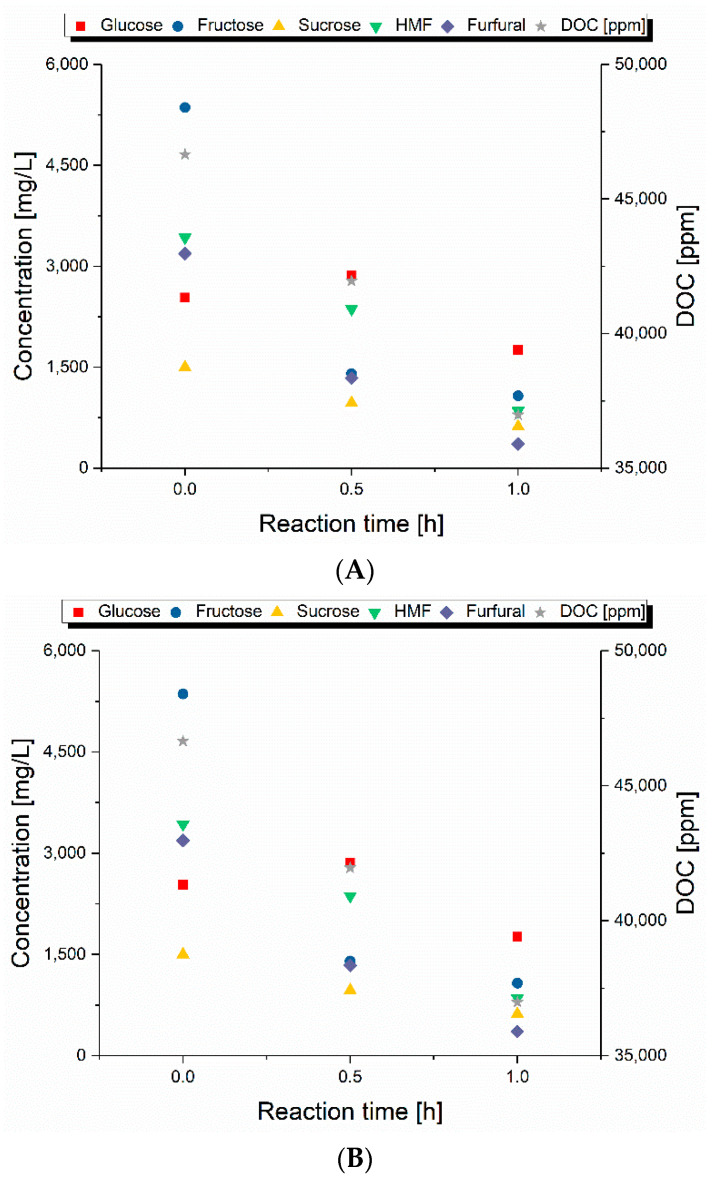
(**A**) Concentration of the sugars, HMF and furfural as well as dissolved organic carbon (DOC); (**B**) concentration of the acids in the process liquid and their pH after HT at 200 °C for different reaction times.

The composition of the liquid samples, obtained at different operating conditions, are shown in Figure 4 and Figure 5 and Supplementary Information. At 160 °C and 0 h reaction, the highest amount of glucose and fructose was obtained, and it was 40 times more than the concentration of HMF and levulinic acid. This meant that the primary products of hydrolysis of carbohydrates were in solution. As proposed by Körner et al., a higher pH leads to a lower production of HMF [6]. With a neutral or weak acidic pH at the beginning of the reaction and due to the low degradation of sugars, the pH of the process liquid at these conditions was consequently the highest of the displayed reactions. Thus, the high amount of sugars was only converted to a low degree to HMF. The increase in the reaction time led to a reduction of the glucose and fructose content in the liquid and an increase in their consecutive products (HMF, formic acid and levulinic acid). Nonetheless, the concentration of HMF was low compared to the projected possibilities mentioned by Körner et al. [6].

**Figure 6:**
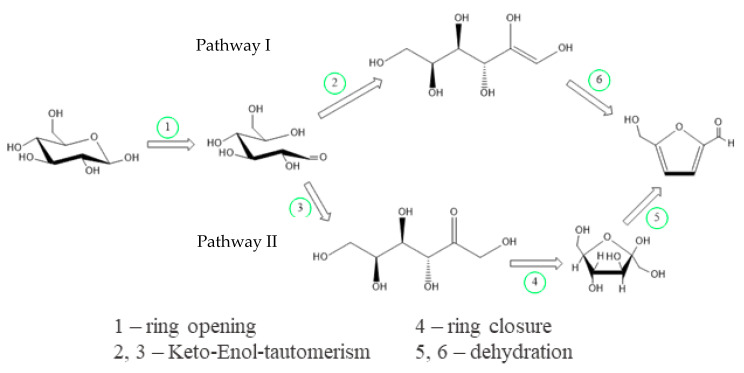
Isomerization of glucose to fructose and subsequent dehydration to form HMF; modified from Ji et al. [62].

The lower amount of fructose compared with glucose found at 160 °C (Figure 4) implied that glucose was converted to HMF via isomerization to fructose by a reversible keto-enol tautomerism, as found by Lobry de Bruyn and Alberda van Ekenstein [58]. On the other hand, Li et al. [59] proposed that the catalytic conversion of glucose to fructose occurs via an enediol intermediate, which then forms fructose and finally builds HMF (Pathway I in Figure 6) or can directly dehydrate to HMF (Pathway II).

This is a critical step for the conversion of polymeric carbohydrates as well as biomass because glucose released from cellulose hydrolysis has a stable pyranose structure, which hampers the dehydration reaction [60].

Aldohexoses like glucose are less reactive; hence, ketohexoses like fructose are superior in producing HMF [6]. This higher reactivity increases the dehydration rate of ketohexoses, which thus react faster than aldohexoses, reducing the formation of unwanted byproducts such as organic acids or a tarry phase (called humins) [61]. As illustrated in Figure 6, fructose can be transformed into HMF with a loss of three water molecules (dehydration step 5) during an acid-catalyzed reaction.

Organic acids like acetic, formic and lactic acids are considered as possible catalysts for the conversion of fructose to HMF [62]. As can be seen from Figure 4 and Figure 5, at higher acid concentrations, the highest amount of HMF was obtained, while it was shown by a study of Körner et al. that the maximum HMF yield was obtained at a pH around 3, even though a lower pH did not necessarily result in a higher HMF yield [7]. Therefore, the conditions of the present study should have had a high selectivity for HMF production, improved by the higher temperature applied. However, the studies mentioned worked with model compounds or solutions, while a real biomass like SPB consists of several other molecules which might affect its conversion behavior. As a result of the conversion of glucose and fructose, higher concentrations of HMF resulted in lower concentrations of both sugars, while the degradation of HMF could be seen at 200 °C for longer reaction times. These results showed that weak carboxylic acids at relatively high concentrations have the required acidity to convert fructose to HMF. This seems promising for developing the process at industrial scale. However, the process worked for fructose, but not for glucose because the concentration of glucose in the solution was still higher than fructose. This meant that the weak Brønsted acidity did not promote glucose isomerization to a high degree. In contrast, a strong Brønsted acidity could enhance the dehydration of glucose to HMF, bypassing the isomerization step [63]. Nevertheless, if the acidity is too high, unwanted reactions can occur such as polymerization between sugars and derivatives and rehydration of HMF to produce levulinic and formic acids [64].

At 200 °C and 0 h reaction time, the highest amount of HMF was obtained; however, the increase of the reaction time to 30 min led to a decrease in the HMF content and an increase in levulinic acid concentration. This effect can be explained by the fact that, under acidic conditions, HMF can be rehydrated to form levulinic and formic acids in aqueous media [65]. Furthermore, self-condensation of HMF and condensation with other compounds leads to the formation of polymeric substances called humins. These humins are built from HMF, and thus consist of furans and hydroxymethyl groups together with aromatic rings [66]. 2,5-Dioxo-6-hydroxyhexanal is supposed to be the key molecule for humin growth formed by rehydration of HMF [67]. However, in this study, no humins were observed after quenching of the HT reaction. This effect was opposite to that observed in previous studies, which reported the activation energies of the formation of humins originating from glucose (51 kJ/mol) and HMF (142 kJ/mol) to be low enough that humins should have been formed [68,69]. Instead of humins, the formation of secondary char preferably occurred. 

## 3. Materials and Methods

### 3.1. Material

Spent sugar beets (SPB) with a water content of 50 wt.% were provided by Südzucker AG. In order to avoid the microbial activity and ensure an equal biomass/water ratio for all the experiments, the biomass was dried at 105 °C for 48 h.

### 3.2. Hydrothermal Treatment

The experimental setup was described in another study of our research group [70]. HT tests were performed in a 250 cm^3^ stainless steel batch autoclave at temperatures of 160 °C and 200 °C and residence times of 0, 30 and 60 min. A reaction time of 0 min meant, after heating up to the desired temperature, the autoclave was directly removed from the oven. For each experiment, 40 g of the dry SPB was mixed with distilled water, keeping a fixed biomass/water ratio of 0.25. After pre-heating, the reactors were held at the desired temperature. The temperature and pressure of the reactor were controlled throughout the whole reaction by a portable data logger (RSG 30 of Endress + Hauser). Once the reaction time was reached, the reactor was quenched in a cold-water bath for 30 min. After the pressure was released, the solids and liquids were separated by vacuum filtration with a Büchner funnel and qualitative filter paper (pore size: 45 µm) from Whatman^®^. The solid residue was dried at 105 °C for at least 24 h and the pH of the separated liquids was recorded. Subsequently, the liquids were immediately stored under freezing conditions (−24 °C). All experiments were done in triplicate.

### 3.3. Characterization of Biomass and Products

#### 3.3.1. Solid Fraction

A pre-dried sample (105 °C for 24 h) was used to determine the fiber composition of the SPB by Fibretherm FT12. The determination of acid detergent lignin (ADL), neutral detergent fiber (NDF) and acid detergent fiber (ADF) was conducted according to Van Soest’s method [71,72]. Cellulose, hemicellulose and lignin, the main components of the fiber, were determined according to Reza et al. [50].

The homogeneity of the samples was ensured by grounding them with a CryoMill from Retsch GmbH for two minutes at 1800 rpm, before the determination of the elemental composition of the SPB and hydrochars. Carbon (C), hydrogen (H), nitrogen (N) and sulfur (S) contents were determined by an elemental analyzer (EuroEA3000 Serie CHNS-O of EuroVector S.p.A) equipped with a thermal conductivity detector (TCD). The oxygen (O) content was calculated by subtraction of the previously determined elemental contents (C, H, N and S) as well as the ash content from 100% [73]. Volatile matter (VM), fixed carbon (FC) and ash content were worked out according to previous studies by Cao et al. [73].

To display the influence of HT on the structures of the surface and to pursue the carbonization of the solid sample, the scanning electron microscope (SEM) FEI Inspect-F, from Queen Mary University in London, was used. A small amount of dry sample was fixed on a carbon tab on the sample holder and sputtered with gold for 90 s. After applying high vacuum, images of the samples were taken.

Solid samples were named as S_XX_YY, where XX denoted the reaction temperature and YY denoted the reaction time (e.g., S_200_0 was the solid product of HT at 200 °C and 0 min, just after heating).

#### 3.3.2. Liquid Fraction

The dissolved organic carbon (DOC) of the liquid samples, previously filtered with a 45 µm Polytetrafluorethylen (PTFE) syringe filter, was determined using the TOC Analyzer 5050A (Shimadzu Scientific Instruments, Columbia, MD, USA). In the next step, the liquids were again filtered with a 13 µm PTFE syringe filter, before the determination of the liquid phase by High-Performance Liquid Chromatography (HPLC) with Shimadzu 20AD. Furthermore, the samples were diluted to 1:10 with distilled water to fit the concentrations of the standard calibration. With an Aminex HPX-87H column, the liquid samples were analyzed for their contents. The eluent used was 4 mM H_2_SO_4_ at a flow rate of 0.65 mL/min and an oven temperature of 25 °C. A more detailed description as well as a discussion of the HPLC method are given in Wüst et al. [74]. All results were directly adjusted with their dilution factor.

Liquid samples were named analogously to the solid samples as L_XX_YY (e.g., L_200_0).

### 3.4. Error Estimation

All samples were prepared with the same procedure; nonetheless, some device-specific errors could have occurred. Balances in the laboratory work were made with an internal uncertainty of 0.005 g for the preparation of the experiment and 0.005 mg for the analysis of the samples. Results of the elemental analysis (EA) were mean values of triplicate measurements of each sample, ensuring that the differences were below 0.5% in-between the measurements. The determination of the composition of the raw spent sugar beets was also performed in triplicate and the uncertainty was 0.05 wt.%. As mentioned in the Supplementary Information in Tables S1 and S2, the uncertainty of the HPLC was below 0.05 g/L due to dilution and internal uncertainty of the device.

## 4. Conclusions 

At two temperatures, the selectivity of the formation of products from hydrolysis and further on from hydrothermal conversion was evaluated. At 160 °C and 0 h, the hydrolysis of biopolymers like cellulose had already started, expressed by the highest sugar contents in the liquid with 25 g/L of glucose and 22 g/L of fructose, while the conversion to degradation products of the sugars was comparatively low. This revealed a higher yield of sugars compared with other biomasses like miscanthus or other lignocellulosic biomass at any time at 160 °C in a batch or at 180 °C in a semi-batch process, each with a catalyst [75,76]. With longer reaction times, the concentration of the sugars decreased until 15 g/L of glucose and 13 g/L of fructose were found. At 200 °C, the trend was opposite for conversion products like acids and HMF, whose yield decreased with longer reaction times. The highest concentration of HMF was obtained at 200 °C and 0 h with 3.4 g/L, which was comparable to the yield of the semi-batch process with a catalyst of lignocellulosic biomasses at the same temperature [75]. Compared with the theoretically possible concentrations using a model compound like fructose, the concentration in the present study did not reach the maximum yet [6]. An optimization of the process parameters (reaction temperature and time) is still necessary to increase the yield of HMF, which should be high with the amount of sugar still present in the spent sugar beets. An optimization of the process parameters is possible by considering the approach of the kinetic modeling of the reactions and optimization of the process parameters reported by Jung et al. [6,7,77]. This will be the subject of future research.

An inclusion of spent sugar beets in biorefineries is possible by considering their use as biofuels. While the raw biomass exhibited an H/C ratio of 1.8, it decreased throughout the reactions, resulting in a ratio of 1.2 for the hydrochar after carbonization for 1 h at 200 °C. This was supported by the increase in the higher heating value up to 22.7 MJ/kg, which was closer to that of subbituminous coal (24.3 MJ/kg) and higher than that of lignite (20.9 MJ/kg) [45].

## Figures and Tables

**Table 1:** Fibers analysis of spent sugar beets (SPB).

Compound	Content [wt.%]
cellulose	17.3
hemicellulose	20.0
lignin	0.6
others	62.1

**Table 2:** Proximate and ultimate analysis of original biomass and hydrochars.

Sample	Proximate Analysis [wt.% db]				Ultimate Analysis Dry [wt.% db]			
	Volatile Matter	Ash	Fixed Carbon	N	C	H	S	O
raw	77.6	5.7	16.6	1.7	42.4	6.2	0.2	43.7
S_160	73.5	5.6	20.9	2.0	45.3	5.8	0.2	41.1
S_160_0.5	72.1	5.8	22.1	2.2	47.8	6.1	0.2	37.9
S_160_1	69.6	5.8	24.2	2.4	52.3	5.8	0.2	33.5
S_200_0	65.8	6.1	28.1	2.3	54.2	5.5	0.2	31.7
S_200_0.5	64.9	5.6	29.5	2.3	55.0	5.5	0.2	31.5
S_200_1	64.5	5.6	30.0	2.1	55.8	5.4	0.2	30.8

**Table 3:** Higher heating value (HHV), energy density (*E_d_*) and total yield (*Y_hydrochar_*) of the hydrochars and raw material.

Sample	HHV [MJ/kg] ^1^	*E_d_*	*Y_hydrochar_* [wt.%]
raw	17.6		
S_160_0	18.4	1.0	63.2
S_160_0.5	19.9	1.1	55.7
S_160_1	21.6	1.2	53.1
S_200_0	22.1	1.3	50.3
S_200_0.5	22.4	1.3	51.6
S_200_1	22.7	1.3	52.8

^1^ HHV calculation by Channiwala and Parikh [51].

## Supplementary Materials

The following are available online. Table S1: Concentration of the sugars in the process liquid, pH and dissolved organic carbon (DOC) after HT; the error was estimated to be 0.05 [g/L] due to dilution errors and high concentrations, Table S2: Concentration of the acids, HMF and furfural in the process liquid after HT; the error was estimated to be 0.05 [g/L] due to dilution errors and high concentrations, Table S3: O/C and H/C ratios of the hydrochars and raw material.
