# Supplementary material for: Hydrothermal Conversion of Spent Sugar Beets into High-Value Platform Molecules
Source: Molecules. 2020 Aug 27;25(17):3914. doi: 10.3390/molecules25173914 (PMC7504806; doi:10.3390/molecules25173914)
Supplement: Supplementary file 1 [file molecules-25-03914-s001.pdf]

Supporting Information

Hydrothermal Conversion of Spent Sugar Beets into High-Value Platform Molecules

Jens Pfersich [1,*], Pablo J. Arauzo [1], Michela Lucian [2], Pierpaolo Modugno [3], Maria-Magdalena Titirici [4], Luca Fiori [2] and Andrea Kruse [1]

[1] Conversion technologies of biobased resources, University of Hohenheim, Garbenstrasse 9, 70599 Stuttgart, Germany; Pabloj.Arauzo@uni-hohenheim.de (P.J.A.); Andrea_Kruse@uni-hohenheim.de (A.K.)

[2] Department of Civil, Environmental and Mechanical Engineering, University of Trento, Via Mesiano 77, 38123 Trento, Italy; Michela.Lucian@unitn.it (M.L.); Luca.Fiori@unitn.it (L.F.)

[3] School of Engineering and Materials Science, Queen Mary University of London, Mile End Road, London E1 4NS, UK; P.Modugno@qmul.ac.uk (P.M.)

[4] Department of Chemical Engineering, Imperial College London, South Kensington, London, SW7 2AZ, UK; M.Titirici@imperial.ac.uk (M.T.)

* Correspondence: Jens.Pfersich@uni-hohenheim.de; Tel.: +49-711-459-24705

Abstract: The growing importance of bio-based products, combined with the desire to decrease the production of wastes, boosts the necessity to use wastes as raw materials for bio-based products. A waste material with a large potential is spent sugar beets, which are mainly used as animal feeds or fertilizers. After hydrothermal treatment, the produced chars exhibited an H/C ratio of 1.2 and a higher heating value of 22.7 MJ/kg, which were similar to that of subbituminous coal and higher than that of lignite. Moreover, the treatment of 25 g/L of glucose and 22 g/L of fructose by heating up to 160 °C led to a possible application of spent sugar beets for the production of 5-hydroxymethylfurfural. In the present study, the maximum concentration of 5-hydroxymethylfurfural was 3.4 g/L after heating up to 200 °C.

Keywords: agro-residues; sugar beets; biomass; hydrothermal carbonization; hydrolysis; sugars; HMF; hydrochar; biorefinery

Table S1: Concentration of the sugars in the process liquid, pH and dissolved organic carbon (DOC) after HT; the error is estimated to be 0.05 [g/L] due to dilution errors and the high concentrations.

**Sample**	**Glucose [g/L]**	**Fructose [g/L]**	**Sucrose [g/L]**	**pH**	**DOC [$^{0}/_{00}$]**
**Raw**	0.2	0.4	3.3	5.98	18.7
**L_160_0**	25.2	22.5	0	3.94	67.2
**L_160_0.5**	17.3	14.3	0.2	3.81	59.3
**L_160_1**	15.0	13.3	0.2	3.72	54.7
**L_200_0**	2.5	5.4	0.2	3.46	46.7
**L_200_0.5**	2.9	1.4	0.1	3.55	42.0
**L_200_1**	1.8	1.1	0.1	3.73	37.0

Table S2: Concentration of the acids, HMF and Furfural in the process liquid after HT; the error is estimated to be 0.05 [g/L] due to dilution errors and the high concentrations.

**Sample**	**Levulinic Acid [g/L]**	**Acetic Acid [g/L]**	**HMF [g/L]**	**Furfural [g/L]**
**Raw**	0	3	0	0
**L_160_0**	0.5	0.4	0.6	0
**L_160_0.5**	0.6	0.5	1.6	0.1
**L_160_1**	1.3	0.1	2.5	0.2
**L_200_0**	3.3	1.4	3.4	0.3
**L_200_0.5**	3.5	1.7	2.4	0.1
**L_200_1**	2.8	1.7	0.9	0

Table S3: O/C and H/C atomic ratios of the hydrochars and raw material.

**Sample**	**O/C**	**H/C**
**Raw**	0.8	1.8
**S_160_0**	0.7	1.5
**S_160_0.5**	0.6	1.5
**S_160_1**	0.5	1.3
**S_200_0**	0.4	1.2
**S_200_0.5**	0.4	1.2
**S_200_1**	0.4	1.2
